# Chronic cyclic vagus nerve stimulation has beneficial electrophysiological effects on healthy hearts in the absence of autonomic imbalance

**DOI:** 10.14814/phy2.12786

**Published:** 2016-05-12

**Authors:** Steven W. Lee, Qinglu Li, Imad Libbus, Xueyi Xie, Bruce H. KenKnight, Mary G. Garry, Elena G. Tolkacheva

**Affiliations:** ^1^ Department of Biomedical EngineeringUniversity of MinnesotaMinneapolisMinnesota; ^2^ Lillehei Heart InstituteUniversity of MinnesotaMinneapolisMinnesota; ^3^ Cyberonics Inc.HoustonTexas

**Keywords:** Arrhythmias, autonomic nervous system, optical mapping, vagus nerve stimulation

## Abstract

Cardiovascular disease degrades the regulatory function of the autonomic nervous system. Cyclic vagus nerve stimulation (VNS) is an already FDA‐approved therapy for drug‐resistant epilepsy and depression, and has been shown to normalize autonomic function and improve objective measures of heart function and subjective measures of heart failure symptoms. However, it remains unclear whether VNS may induce negative effects in patients with potentially healthy hearts where VNS can be used for epileptic patients. Hence, this study aims to investigate the effects of VNS on the hearts of healthy rats with normal autonomic balance. Sprague–Dawley rats were implanted with stimulators and randomized to either Sham or VNS groups. Rats in VNS group received 10 weeks of chronic intermittent VNS via stimulation of the right cervical vagus nerve. Echocardiography was performed at Baseline (prior to VNS), Week 2, and Week 9. After 10 weeks, high‐resolution optical mapping was performed in ex vivo perfused hearts to evaluate the electrophysiological remodeling that occurs in the heart as a result of the VNS therapy. Chronic VNS modified the electrophysiological properties of healthy rat hearts by reducing the action potential duration at 50% (APD_50_) and 80% (APD_80_) repolarization. Chronic VNS also affected the restitution properties of the heart at the APD_50_ level and increased myocardial conduction velocity (CV). VNS did not induce any significant changes to ventricular ejection fraction (EF) and spatial dispersion of APD, thus indicating that VNS did not negatively affect cardiac function. VNS also reduced the susceptibility to ventricular arrhythmias (ventricular fibrillation [VF] and ventricular tachycardia [VT]) during ex vivo programmed electrical stimulation. In summary, chronic application of cyclic VNS induces changes to the electrophysiological properties of healthy rat hearts. The observed decrease in APD and increase in CV suggest that the beneficial effects of VNS do not require the presence of existing autonomic imbalance.

## Introduction

Vagus nerve stimulation (VNS) is an approved clinical therapy for drug‐refractory epilepsy and depression, and more than 130,000 VNS therapy systems have been implanted since 1995 (Shuchman 2007). In the past decade, several preclinical and clinical studies have demonstrated the beneficial effects of VNS on cardiovascular diseases via parasympathetic nervous system activity modulation (De Ferrari and Schwartz 2011; Sabbah 2011; Beaumont et al. 2015).

The autonomic nervous system consists of two distinct branches: the parasympathetic and sympathetic nervous systems. The balance between the two systems plays a significant role in regulating cardiovascular functions and specifically, the coordination of the electromechanical function of the heart, leading to optimized cardiac output under a variety of environmental and metabolic stressors. Heart diseases such as chronic heart failure (HF) and hypertension (HTN) are associated with autonomic dysregulation characterized by a sustained increase in sympathetic drive and withdrawal of parasympathetic activity (Bibevski and Dunlap 2011; Schwartz and De Ferrari 2011). VNS has emerged as a promising therapy to treat cardiovascular diseases by its proposed mechanism to correct this autonomic imbalance (Annoni et al. 2015; Beaumont et al. 2015; Li et al. 2004; Xueyi Xie et al. 2014; De Ferrari and Schwartz 2011; Premchand et al. 2015; Schwartz and De Ferrari 2009).

Nonetheless, despite the extensive research and advances that have been made in recent years to support the concept of electrical VNS as a therapeutic approach, very little is known about the effects of chronic VNS on healthy hearts. While it has always been known that parasympathetic vagal nerve fibers densely innervate the atria and the sinoatrial (SA) and atrioventricular (AV) nodes, it was initially believed by many that vagal fibers only sparsely innervate the ventricles (Coote 2013). However, other studies have challenged this supposition and suggest that the vagal fibers, in fact, play a greater role in ventricular function than initially believed (Armour et al. 1997; Randall et al. 2003; Ulphani et al. 2010; Coote 2013).

As a result of this finding, it is important to gain a better understanding and fully characterize how stimulating the vagus nerve may affect the normal heart when the autonomic nervous system is in balance. In addition, as applies to any novel therapy, testing in normal, healthy controls is essential to understanding the mechanisms of action of VNS. Most importantly, it makes it feasible to evaluate any potential negative effects the therapy may have without the interferences of other confounding factors. Moreover, VNS is FDA‐approved to treat epileptic patients; however, the electrophysiological side effects of VNS on their potentially healthy hearts are not well characterized. Hence, the main objective of this novel study is to investigate the effects of chronic, intermittent VNS on the electrophysiological properties of healthy rat hearts.

## Methods

### Animal preparation

All animal experiments were approved by the University of Minnesota Animal Care and Use Committee and were conducted in accordance with both the Institutional and National Institute of Health Guidelines for the Care and Use of Laboratory Animals.

Male Sprague–Dawley rats (*n* = 9, 250–300 g, Charles River Laboratories, Wilmington, MA) were randomized into two groups: Sham (*n* = 6) and VNS (*n* = 3). Sham group animals were implanted with nonfunctional vagus nerve stimulators, and VNS group animals were implanted with functional vagus nerve stimulators (Demipulse Model 103, Cyberonics Inc., Houston, TX). All animals were maintained and monitored for a total of 10 weeks. Rats were placed in a quiet, temperature‐ and humidity‐controlled room with a 12:12 h light–dark cycle. Food and water were available ad libitum.

### VNS stimulator implantation

The VNS pulse generator was implanted subcutaneously on the lower back of the rats as described previously (Xueyi Xie et al. 2014; Annoni et al. 2015). During the surgery, rats were anesthetized with isoflurane (5% for induction; 2% or 3.5% for maintenance) in oxygen (2 L/min for induction, 1 L/min for maintenance). The rats’ body temperature was maintained at 37°C on a temperature‐controlled surgical table. The back and the neck of the rat were shaved, and the right cervical vagus nerve and common carotid artery bundle were isolated from the surrounding tissue through a small incision on the neck. The custom 1.5 mm diameter helical lead bipolar cuff electrodes were placed around the carotid artery bundle containing the vagus nerve. The therapy group received continuous cyclic low‐level VNS for 10 weeks. Low‐level VNS parameters were set as follows: stimulation frequency of 20 Hz, pulse width of 500 *μ*sec, and stimulation current of 1.0 mA. The pulse generator was programmed to deliver continuously cyclic VNS: 7 sec ON and 66 sec OFF. The effectiveness of VNS and its effect on acute and chronic changes in heart rate have been evaluated in previous studies (Annoni et al. 2015; Xueyi Xie et al. 2014).

### Echocardiography

Echocardiography was performed in both VNS (*n* = 3) and Sham (*n* = 2) rats prior to VNS implantation surgery (Baseline), two weeks after surgery (Week 2), and one week prior to killing (Week 9). Anesthesia was induced with 2% isoflurane gas in 100% oxygen, and transthoracic echocardiography (VisualSonics, VEVO‐770 with 700‐Series RMV Scanhead Probe, Toronto, Canada) was performed. Echocardiographic M‐mode images of the left ventricle (LV) were obtained using a parasternal short‐axis view at the middle of papillary muscle level. Left ventricular end diastolic (LVEDV) and systolic (LVESV) volumes were measured and used to calculate the percentage of LV ejection fraction (EF), an index of LV function, for each animal using the following equation: EF = ((LVEDV − LVESV)/LVEDV) × 100%.

### High‐resolution optical mapping

At the end of 10 weeks, VNS (*n* = 3) and Sham (*n* = 4) rats were killed and hearts were quickly extracted through a thoracotomy. Immediately upon removal, the hearts were immersed in cold cardioplegic solution (in mmol/L: glucose 280, KCl 13.44, NaHCO_3_ 12.6, and mannitol 34). The aorta was then quickly cannulated and perfused (retrograde) with warm (37 ± 1°C) oxygenated Tyrode's solution (in mmol/L: NaCl 130, CaCl_2_ 1.8, KCl 4, MgCl_2_ 1.0, NaH_2_PO_4_ 1.2, NaHCO_3_ 24, glucose 5.5, and pH 7.4). The hearts were immersed in a chamber and superfused with the same Tyrode's solution.

After 30 min of stabilization, voltage‐sensitive dye (di‐4‐ANEPPS, 5 *μ*g/mL; Molecular Probes) was added to the perfusate. Two 532 nm diode continuous green lasers (SDL‐532‐1000T, Shanghai Dream Lasers Tech, Shanghai, China) were used to illuminate both the right ventricles (RV) and LV surfaces of the heart. Fluorescence signals from more than 80% of total ventricular surface were captured with two 12‐bit charge‐coupled device (CCD) cameras (DALSA, Waterloo, Canada), that ran synchronously at 600 frames per second with 64 × 64 pixel resolutions. Blebbistatin (10–15 μmol/L) was added to the Tyrode's solution to stop heart contractions and reduce motion artifacts (Smith et al. 2012).

Hearts were paced periodically. An external stimuli (5 msec duration, twice the activation threshold) were applied to the base of the RV at progressively decreasing basic cycle length (BCL) from 200 msec in steps of 20 msec until BCL reached 80 msec or until ventricular tachycardia (VT) or fibrillation (VF) was initiated (Annoni et al. 2015). If and only when VF/VT was not induced during periodic pacing, a burst pacing protocol was used. At each BCL, 40 stimuli were applied to reach steady state. Optical mapping movies were recorded at steady state of each pacing BCL from both LV and RV epicardial surfaces of the heart. The background fluorescence was subtracted from each frame, and spatial (3 × 3 pixels) and temporal (5 pixels) convolution filters were used.

### Parameter measurements

Both LV and RV optical mapping movies were used for data analysis. No significant differences were found between data from the two ventricles, and therefore, data was pooled for analysis.

#### Action potential duration measurements

Optical action potential duration was measured at both 50% and 80% repolarization (APD_50_ and APD_80_, respectively), and two‐dimensional (2D) APD maps were constructed to reveal the spatial distribution of APDs on the epicardial surface of the heart. Mean APD was obtained at different BCLs by averaging APDs from all pixels.

#### Conduction velocity measurements

Local conduction velocity (CV) was calculated as described previously (Smith et al. 2012; Annoni et al. 2015). Specifically, the distributions of activation times (measured at (dV/dt)_max_) for the spatial regions of 5 × 5 pixels were fitted with the plane, and gradients of activation times $g_{x}$ and $g_{y}$ were calculated for each plane along the x and y axes, respectively. The magnitude of the local CV was calculated for each pixel as ${\left(g_{x}^{2}+g_{y}^{2}\right)}^{-1/2}$.

#### APD heterogeneity measurements

The spatial dispersion of APD or dispersion of repolarization at both 50% and 80% repolarization was estimated based on the heterogeneity index, *μ* (Smith et al. 2012; Annoni et al. 2015):

$$\mu =\frac{{\text{APD}}^{95}-{\text{APD}}^{5}}{{\text{APD}}^{50}}$$

where APD^95^ and APD^5^ represent the 95th percentile and 5th percentiles of the APD distribution, respectively, and APD^50^ is the median of APD distribution.

### Statistical analysis

All data are presented as means ± SE. Statistical comparisons of APD, *μ*, and echocardiography data between Sham and VNS groups were performed using one‐way ANOVA test (Origin Software, Northampton, MA). Statistical comparison of echocardiographic assessment among Baseline, Week 2, and Week 9 were performed using a paired Student's t‐test. Comparison of CV was performed using a paired *t*‐test. Values of *P* < 0.05 were considered to be statistically significant.

## Results

**Figure 1:**
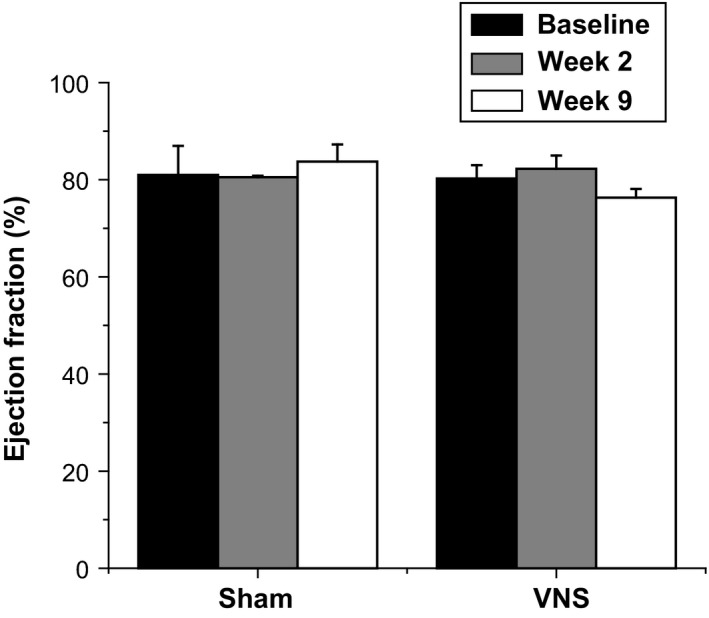
Effects of long‐term intermittent VNS on left ventricular (LV) function. Mean ejection fraction (EF) (%) measurements from Sham and VNS rats for Baseline, Week 2, and Week 9.

To determine both the in vivo acute and chronic effects of VNS on cardiac performance in the healthy animals, LV function was assessed using echocardiography at both Weeks 2 and 9 after VNS implantation. As presented in Figure 1, VNS rats exhibited similar EF as compared to Sham rats throughout the duration of the study. Furthermore, EF did not change significantly over time in both Sham and VNS rats. Measurements of fractional shortening were also made, and no significant differences were observed (data not shown).

**Figure 2:**
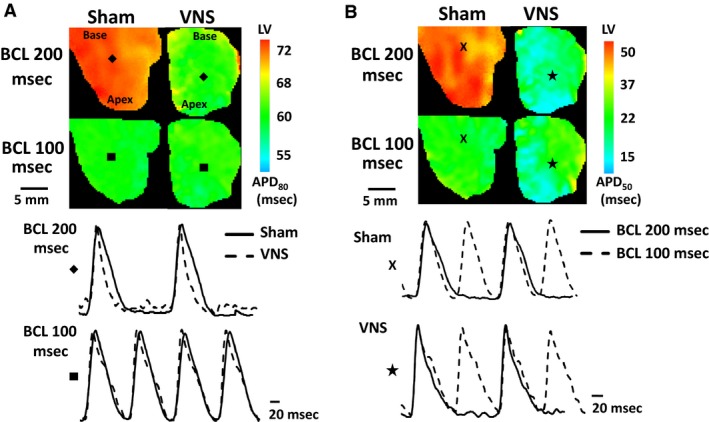
Representative left ventricular (LV) action potential duration (APD) maps at basic cycle length (BCL) = 200 msec and 100 msec. (A) Examples of APD_80_ maps with action potential traces from pixels “
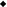
” and “
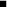
” for BCLs = 200 msec and 100 msec, respectively. (B) Examples of APD_50_ maps with representative action potential traces from pixels “
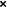
”and “
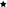
”for Sham and VNS, respectively.

Ex vivo optical mapping was used to probe action potential conduction and restitution dynamics in order to determine how long‐term intermittent VNS modulates the basic electrophysiological properties of the healthy myocardium. Changes in APD were measured during periodic pacing at gradually decremented BCLs. Figure 2A and B (top panels) show examples of 2D APD_80_ and APD_50_ maps, respectively, obtained at a BCL of 200 msec and 100 msec for both Sham and VNS‐treated rats. The lower panels show corresponding traces of action potentials from a representative single pixel. Figure 2A bottom panel compares VNS versus Sham traces at the same BCLs. APD from the VNS group is shorter than APD of the Sham group for BCL 200 msec, but not for 100 msec. Figure 2B compares both VNS and Sham action potential traces that were recorded at different BCLs.

**Figure 3:**
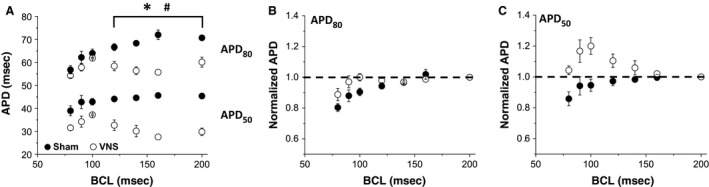
Effects of long‐term intermittent VNS on APD. (A) Mean APD_50_ and APD_80_ values for both Sham and VNS at different BCLs. Normalized (B) APD_80_ and (C) APD_50_ values to BCL 200. *^, #^Statistical significance (*P* < 0.05) between Sham and VNS for APD_80_ and APD_50,_ respectively.

The change in APD of all VNS and Sham rats is shown in Figure 3A for different BCLs. At higher BCLs (between 200 msec and 120 msec), the APDs of VNS rats (both APD_50_ and APD_80_) were significantly lower than those of Sham rats. For instance, at BCL 200 msec, the APD_80_ and APD_50_ values for Sham rats were 70.69 ± 0.34 msec and 45.39 ± 0.84 msec, respectively. After VNS treatment, the APD_80_ and APD_50_ values of VNS rats were both significantly (*P* < 0.05) reduced to 60.12 ± 2.16 msec and 29.81 ± 1.59 msec, respectively. This APD shortening effect disappeared at lower BCLs (between 100 msec and 80 msec). At BCL 100 msec, the APD_80_ values for Sham and VNS rats were 63.98 ± 1.82 msec and 61.87 ± 0.22 msec (*P *= NS), respectively. Furthermore, the APD_50_ values for Sham and VNS rats were 42.89 ± 1.42 msec and 37.22 ± 0.28 msec (*P *= NS).

Calculation of the restitution properties of the heart (i.e., the change of APD as BCL decreases) showed that the APD of VNS rats (both APD_80_ and APD_50_) did not monotonically decrease as BCL decreased, in contrast to APDs of Sham rats (Fig. 3A). Therefore, it was not feasible to fit the VNS data to a single curve and accurately calculate the slope of restitution curve for VNS rats. Hence, we used an alternative approach, in which APD values at different BCLs were normalized to the APD value at BCL 200 msec for each individual rat. The mean normalized APD data for both Sham and VNS rats for APD_80_ and APD_50_ are shown in Figure 3B and C, respectively. Note that APD_80_ decreased as BCL decreases, showing normal restitution properties, for both VNS and Sham rats. However, the behavior of APD_50_ is different: the monotonic decrease in APD was observed for Sham, but a biphasic change in APD, with initial increase and subsequent decrease, was observed in VNS rats. These data suggest that VNS affects the dynamic behavior of the heart for APD_50_, but not for APD_80_.

**Figure 4:**
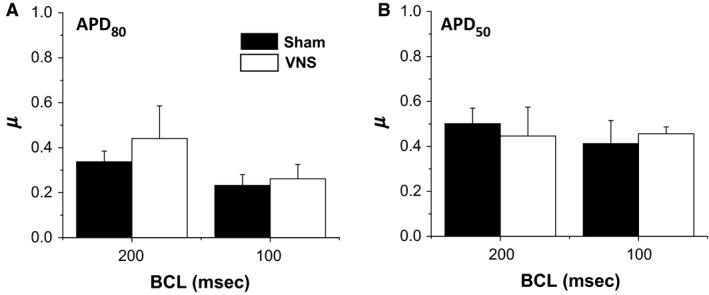
Effects of VNS on spatial dispersion of APD (*μ*). Mean *μ* values for (A) APD_80_ and (B) APD_50_ for BCLs = 200 msec and 100 msec.

To determine whether VNS affected the spatial dispersion of APD, we calculated the heterogeneity index *μ* at different BCL values for both APD_50_ and APD_80_. The *μ* values calculated for BCL 200 msec and 100 msec are shown in Figure 4A and B, demonstrating no significant differences in *μ* values between VNS and Sham at different BCLs. The values are similar for other BCLs (data not shown).

**Figure 5:**
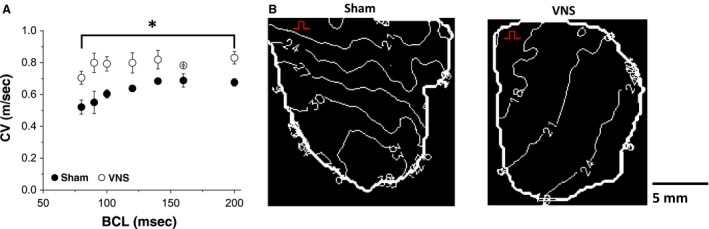
Effects of VNS on conduction velocity (CV). (A) Mean CV values at different BCL values. (B) Representative examples of LV action potential activation maps for the epicardial surfaces of Sham and VNS rats at BCL = 100 msec. Isochrones for activation time maps are shown 3 msec apart. The red marker denotes pacing site. *Statistical significance (*P* < 0.05).

To investigate the effect of VNS on action potential propagation in the ventricular myocardium surfaces, we constructed activation time maps for both Sham and VNS groups at various BCLs. Representative examples of 2D activation time maps at BCL 100 msec is shown in Figure 5B. These maps show normal propagation in both VNS and Sham rats. Figure 5A shows mean CV data at different BCLs. Chronic VNS significantly increased the mean CV of impulse propagation in the ventricles for all BCLs (*P* < 0.05). Specifically, by 23% for BCL 200 msec (from 0.676 ± 0.024 m/sec to 0.830 ± 0.039 m/sec) and by 31% for BCL 100 msec (from 0.604 ± 0.026 m/sec to 0.792 ± 0.045 m/sec).

**Figure 6:**
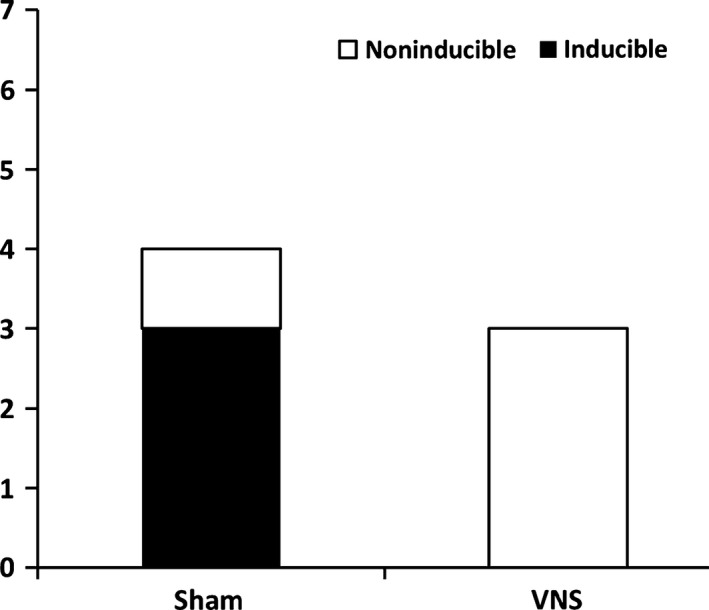
Quantification of the number of rats exhibiting ex vivo ventricular fibrillation (VF) and tachycardia (VT) episodes during programmed stimulation.

The anti‐arrhythmic effects of VNS on healthy hearts were assessed by measuring the inducibility of the hearts to cardiac arrhythmias during ex vivo programmed electrical stimulation. As shown in Figure 6, sustained VF/VT was observed in 3/4 Sham rats, whereas none (0/3) of the VNS rats developed VF or VT.

## Discussion

In this study, we investigated the effects of chronic, cyclic VNS on healthy rat hearts. The main findings of this paper are as follows: (1) 10 weeks of intermittent VNS produced beneficial electrophysiological changes to the heart, including reduction of APD at larger BCLs and an increase of CV at all BCLs; (2) VNS did not induce any detrimental effects to the heart: neither affecting the LV function of the heart verified via echocardiography nor increasing the spatial dispersion of APD (*μ*); (3) VNS significantly affected the restitution properties of the heart at the APD_50_, but not at the APD_80_, levels; (4) VNS had anti‐arrhythmic effects that has been previously only reported for diseased hearts (Annoni et al. 2015; Beaumont et al. 2015). These results support the beneficial effects of VNS even in the absence of autonomic imbalance.

It is universally accepted that the autonomic nervous system, consisting of both the parasympathetic and sympathetic systems, plays an important role in regulating the function of the heart (Hirsch et al. 1987; De Ferrari and Schwartz 2011; Schwartz and De Ferrari 2011). In the last decades, an abundance of experimental and clinical evidence has shown that cardiovascular diseases are accompanied by an imbalance in the vagal‐sympathetic outflow to the heart. It has been demonstrated that the autonomic balance can be potentially restored through the electrical stimulation of the vagus nerve (Li et al. 2004; Annoni et al. 2015; Premchand et al. 2015); however, not much is known about the effects of parasympathetic simulation via VNS on healthy hearts where the sympathetic and parasympathetic nervous systems are in balance. This can be attributed to the early major belief that vagal fibers have sparse innervations and play only a limited role in ventricles. Since, however, histochemical evidence from several studies has now convincingly questioned this viewpoint (Randall et al. 2003; Hoover et al. 2004; Ulphani et al. 2010; Coote 2013), it becomes increasingly important to gain a better and clearer understanding of the effects of VNS. This is especially true since VNS is now emerging as a promising neuromodulation therapy for HF due to positive results from multiple clinical trials, such as the Autonomic Neural Regulation Therapy to Enhance Myocardial Function in Heart Failure (ANTHEM‐HF) (Premchand et al. 2015).

Slowing of myocardial CV may increase the risk of cardiac arrhythmias (Gaztañaga et al. 2012). This is because a decrease in CV allows the creation of smaller wavelengths, thus facilitating the initiation and maintenance of reentry subsequent to creation of functional conduction block. Our data suggest that VNS is able to moderately increase CV. This increase can be attributed to the increase or further preservation of the gap junction protein connexin‐43 (Cx‐43) expression in the myocardium (Sabbah 2011). It has been shown in previous studies that VNS is able to preserve the expression of Cx‐43 in a rat model of MI (Wu and Lu 2011).

Prolongation of the ventricular APD is a hallmark of HF (Shah et al. 2005). In cases of HF, APD prolongation allows for the myocardium to preserve its contractility by prolonging calcium channel opening. However, this compensatory APD prolongation also causes intracellular calcium overload, increasing the propensity for triggered arrhythmias and increased T‐wave alternans (Shimizu and Antzelevitch 1999). Furthermore, this significant APD prolongation is typically associated with an increase in dispersion of repolarization (Wang and Hill 2010). In other words, increases in both of these parameters have been shown to be able to create an arrhythmogenic substrate. Our results suggest that long‐term intermittent VNS therapy decreases APD during programmed electrical stimulation and does not induce spatial APD dispersion, thereby rendering the hearts less vulnerable to inducible tachyarrhythmia as shown in Figure 6.

Indeed, in the settings of HF with the characteristics of APD prolongation and CV slowing which may promote re‐entrant arrhythmias, our observed effects of APD shortening and CV acceleration may be able to explain the beneficial effects of VNS. This, however, may be different in healthy hearts. In fact, APD shortening induced by activation of acetylcholine‐activated potassium channels has been widely used as an arrhythmia model (Machida et al. 2011; Cho et al. 2014), and CV slowing has been an anti‐arrhythmic feature of Class I anti‐arrhythmic drugs (Cha et al. 1996; Kirchhof et al. 1998). APD shortening and CV acceleration have opposite effects on the wavelength for reentry, that is, wavelength is the product of APD and CV which both are measured at a particular BCL. The wavelength can be used as an indicator of arrhythmia propensity in structurally normal hearts (Smeets et al. 1986; Pandit and Jalife 2013).

Finally, our results suggest that VNS may affect the restitution properties of the heart, specifically at the APD_50_ level, which may suggest an increase in intracellular calcium handling. Our observations seem to support recent findings where VNS treatment activates the ryanodine receptor 2 (RyR2) channels (Li et al. 2015) and increases both sarcoplasmic reticulum Ca^2+^ ATPase (SERCA2) and sodium–calcium exchanger (NCX1) protein expressions in a chronic HF rat model (Zhang et al. 2015). The activation of RyR2 channels increases calcium release from the sarcoplasmic reticulum (SR) (systole) which SERCA2 will pump the calcium back to the SR (diastole). Taken together, VNS is able to increase the expression levels of RyR2 and SERCA2; thus, enhancing excitation contraction coupling and improving cardiac pump function. Nonetheless, additional experiments will need to be performed to specifically determine which myocyte membrane ion channels are up‐ or downregulated by chronic VNS.

In conclusion, we demonstrate that the administration of chronic intermittent vagus nerve stimulation (VNS) induces electrophysiological changes to a normal, healthy rat heart; thus, rendering the hearts less vulnerable to inducible arrhythmias. These results also suggest that VNS does not require the existence of autonomic imbalance to generate its positive effects.

## Limitations

While we were able to demonstrate the effects of VNS on the electrophysiological properties of the healthy myocardium, no histochemical results from the ventricles were performed in parallel to confirm and/or investigate mechanisms. Another limitation is that an animal group with age‐matched controls, that is, that did not have an implanted stimulator and electrode, was not included in this study. However, since the objective of our study was to directly investigate the effects of VNS, we removed the confounding factor of surgical procedure effects on electrophysiology by implanting nonfunctional stimulators in our Sham group. Hence, not including such a control group will not affect the interpretation of our results. The conclusions of our study are also limited by the small sample size of rats used.

## Conflict of Interest

Drs. Imad Libbus and Bruce H. KenKnight are employees of Cyberonics Inc.
